# A Systematic Ex-Vivo Study of the Anti-Proliferative/Cytotoxic Bioactivity of Major Olive Secoiridoids’ Double Combinations and of Total Olive Oil Phenolic Extracts on Multiple Cell-Culture Based Cancer Models Highlights Synergistic Effects

**DOI:** 10.3390/nu15112538

**Published:** 2023-05-29

**Authors:** Aikaterini Papakonstantinou, Petrina Koumarianou, Panagiotis Diamantakos, Eleni Melliou, Prokopios Magiatis, Haralabia Boleti

**Affiliations:** 1 Intracellular Parasitism Laboratory, Microbiology Department, Hellenic Pasteur Institute, 11521 Athens, Greece; a.papak@pasteur.gr (A.P.); pkoumar@gmail.com (P.K.); 2 Laboratory of Pharmacognosy and Natural Products Chemistry, Department of Pharmacy, National and Kapodistrian University of Athens, Panepistimioupolis Zografou, 15771 Athens, Greece; pdiam@pharm.uoa.gr (P.D.); emelliou@pharm.uoa.gr (E.M.); 3 World Olive Center for Health, Imittou 76, 11634 Athens, Greece

**Keywords:** olive phenols combinations, secoiridoid derivatives, anti-proliferative efficacy, cytotoxic efficacy, anticancer properties, olive oil phenolic extracts, human cancer cells, oleocanthal

## Abstract

Several individual olive oil phenols (OOPs) and their secoiridoid derivatives have been shown to exert anti-proliferative and pro-apoptotic activity in treatments of human cancer cell lines originating from several tissues. This study evaluated the synergistic anti-proliferative/cytotoxic effects of five olive secoiridoid derivatives (oleocanthal, oleacein, oleuropein aglycone, ligstroside aglycone and oleomissional) in all possible double combinations and of total phenolic extracts (TPEs) on eleven human cancer cell lines representing eight cell-culture-based cancer models. Individual OOPs were used to treat cells for 72 h in half of their EC_50_ values for each cell line and their synergistic, additive or antagonistic interactions were evaluated by calculating the coefficient for drug interactions (CDI) for each double combination of OOPs. Olive oil TPEs of determined OOPs’ content, originating from three different harvests of autochthonous olive cultivars in Greece, were evaluated as an attempt to investigate the efficacy of OOPs to reduce cancer cell numbers as part of olive oil consumption. Most combinations of OOPs showed strong synergistic effect (CDIs < 0.9) in their efficacy, whereas TPEs strongly impaired cancer cell viability, better than most individual OOPs tested herein, including the most resistant cancer cell lines evaluated.

## 1. Introduction

The chemopreventive properties of olive oil in cancer have been associated with its unique phenolic compounds represented by hydroxytyrosol and tyrosol and mainly by their secoiridoid derivatives oleuropein aglycone, ligstroside aglycone, oleacein and oleocanthal [1,2,3,4,5,6,7]. Moreover, the phenols found in olive oil have well-established beneficial effects on human health and metabolism [8,9,10,11]. Secoiridoids are a group of compounds found in several plant families, such as *Oleaceae, Valerianaceae, Gentianaceae* and *Pedialaceae.* They are abundant in the European olive tree (*Olea europaea* L.) and they comprise the main bioactive polyphenols in olive oil and drupes [12,13,14,15,16,17]. The majority of secoiridoid phenolic derivatives in olive oil come from oleuropein and ligstroside, the major secoiridoids in the olive fruit. Extra virgin olive oil (EVOO) also contains the stable enolic form of oleuropein and ligstroside aglycone secoiridoids, named oleomissional and oleokoronal. The structures of the latter have been elucidated by Diamantakos et al. 2015 [18]. Oxidation products of oleocanthal and oleacein are present in fresh oils in very low concentrations. The oleocanthalic acid concentration increases with storage time, while that of oleocanthal decreases [19].

In a recently published study [20], six individual olive oil phenols (OOPs) were analyzed for their anticancer efficacy in vitro. The study demonstrated their anti-proliferative and pro-apoptotic action and the EC_50_ values for oleuropein aglycone (3,4, DHPEA-EA), ligstroside aglycone (p-HPEA-EA), oleocanthal (p-HPEA-EDA), oleacein (3,4, DHPEA-EDA) and oleomissional on sixteen tumor cell lines from eight different cancer tissue origins were calculated. This study demonstrated that the efficacy of each individual OOP tested varied amongst the different cancer cell lines, suggesting that they may act via different mechanisms. Moreover, OOPs’ final effects when obtained as dietary components may be influenced by their interactions with the other components of olive oil. Investigations of the anticancer efficacy of other plant-derived polyphenols have shown that combinations of them exhibit biological activities different from those detected with individual phenolic compounds [21,22,23,24]. Polyphenols are known to interact additively, synergistically or antagonistically with each other, with other food components as well as with synthetic drugs [25,26]. Therefore, their bioactivity can be further enhanced by combining them with chemically similar or different compounds with which they may have a synergistic effect [27]. All the above highlight the importance of investigating the efficacy of OOPs to produce anti-proliferative/cytotoxic effects when used in double combinations so as to detect synergies between pairs of individual OOPs. Of equal importance is to study their efficacy in the presence of the other OOPs in olive oil total phenolic extracts (TPEs).

The current study presents results on the anti-proliferative/cytotoxic efficacy of all possible double combinations of five olive oil secoiridoids. Its advantage is that all OOPs tested were provided by the same laboratory specialized in the isolation and characterization of OOPs [18,28,29], which ensured reproducibility of results. Several of the OOPs’ double combinations with promising anticancer efficacy in different types of cancer cells are highlighted. The observed synergy between the analyzed OOPs is very important as it allows the use of low, active concentrations for each substance and reduced toxic effects on normal cells. In addition, this study evaluated the cytotoxic activity of TPEs as compared to isolated OOPs. Three different olive oil TPEs varying in their OOP composition were analyzed for their anti-proliferative/cytotoxic activity on eleven cancer cell lines from eight cancer types, with results revealing interesting synergies and efficacies of less-studied OOPs. These results are of great interest in the search for new chemopreventive agents that are less toxic than the ones used in conventional anticancer chemotherapies to be used as adjuvants in anticancer therapy.

## 2. Materials and Methods

### 2.1. Chemicals

Thiazolyl blue tetrazolium bromide (MTT, M5655) and dimethyl sulfoxide for cell culture (DMSO, D2650) were from Sigma-Aldrich (Darmstadt, Germany).

The olive plant phenolic compounds used in this study (Table S1) were isolated and purified by the methodology described in Papakonstantinou et al., 2022 [20]. The TPEs were produced as presented in the section below.

### 2.2. Preparation and Analysis of Olive Oil Total Phenolic Extracts (TPEs)

Three different olive oils were selected after the screening of hundreds of olive oil samples available in the collection of the Laboratory of Pharmacognosy. The samples were selected based on the different ratios among the main phenolic ingredients of olive oil as observed through the ^1^H-NMR analysis [28,29,30]. The extracts were prepared after dilution of the olive oil (5 g) with cyclohexane (15 mL) and extraction with MeOH/H_2_O (80:20) (20 mL). The hydroalcoholic mixture was obtained and evaporated to dryness. Special care was taken to remove all traces of solvents before use in biological testing. The analysis of extract was performed by qNMR using a Bruker DRX400 as previously described [30].

### 2.3. Cell Lines, Culture Conditions and Treatment Protocols with OOPs

The cell lines used in this study were obtained from different sources as described in Table S2. High glucose (4500 mg/L), Dulbecco modified Eagle medium (DMEM) (LM-D1109, Biosera, Nuaillé, France) supplemented with 10% (*v*/*v*) fetal bovine serum (FBS) (FB-1001, Biosera), 1mM sodium pyruvate (SH30239.01, HyClone, Logan, UT, USA) and non-essential amino acids (SH30238.01, HyClone) was used as a complete medium for the maintenance in culture of the three breast cell lines (i.e., MDA-MB 231, MCF-7 and SK-BR-3) as well as for the A2058, NHDF and AGS cell lines. The HaCaT cells were maintained in the same above-described complete medium without supplementation with sodium pyruvate and non-essential amino acids. This medium contains 4 mM L-glutamine, as suggested for the medium supplements in the CLS company instructions (https://cls.shop/HaCaT/300493?proid=800, accessed on 25 May 2023). Concerning the PANC-1 cell line, the culture medium used was a high glucose DMEM (SH30081.01, HyClone) with 10% (*v*/*v*) FBS (FB-1001, Biosera) supplemented with 2 mM glutamine (SH 30034.01, HyClone). RPMI GlutaMAX (LMR 1640/500, Gibco, Waltham, MA, USA) supplemented with 10% (*v*/*v*) FBS and 1 mM sodium pyruvate was the complete culture medium for the SK-MEL-28 and H1299 cell lines, while HT-29 cells were maintained in the above-mentioned complete medium without the addition of sodium pyruvate. The Hela and HepG2 cell lines were cultured in DMEM GlutaMAX with 10% (*v*/*v*) FBS supplemented with 1 mM sodium pyruvate. Finally, the non-tumorigenic breast cancer cell line MCF 10A was maintained in a Mammary Epithelial Cell Growth Medium with 5% (*v*/*v*) horse serum (HS) (26050088, Gibco) supplemented with 0.004 mL/mL BPE, 10 ng/mL EGF, 5 μg/μL insulin and 500 ng/mL hydrocortisone (C21110, Promega). The antibiotics penicillin (100 U/mL; LM-A4118, Biosera) and streptomycin (100 μg/mL; LM-A4118, Biosera) were added in all the culture media and cells were maintained in a humidified atmosphere at 37 °C with 5% (*v*/*v*) CO_2_. Trypsinization of cells for subculturing was performed with one-step washing with Dulbecco Phosphate Buffered Saline (PBS) *w*/*o* Calcium and Magnesium (LM-S2041, Biosera) and incubation (~5 min, 37 °C) with trypsin-EDTA 0.05% (*w*/*v*) in PBS without (*w*/*o*) Calcium and Magnesium with Phenol Red (LM-T1705, Biosera).

### 2.4. Treatment of Cell Lines with TPE and Double OOP Combinations

For the treatment experiments, the methodology described previously by Papakonstantinou et al. 2022 was followed with same modifications. More specifically, for double combinations of OOPs, 0.25 μL of a 1000× stock of each tested compound in DMSO was added directly into each well in a 250 μL final volume of medium and the solution was gently mixed by pipetting. The concentration used in the experiments with the combination of OOPs was ½ of the EC_50_ value for each compound in the particular cell line [20]. Cells treated only with the vehicle compound [DMSO 0.2% (*v*/*v*)] were used as control groups. Concerning the evaluation of the anti-proliferative/cytotoxic properties of TPEs, three extracts of different phenolic composition were used (Table S3). An appropriate amount of dry extract was dissolved in DMSO to a final concentration of 50 mM (stock solution) based on the OOP with the highest concentration. The stock solution was further used to prepare various TPE concentrations in DMSO. For the determination of the EC_50_ values for each TPE, five different concentrations were evaluated and the cell viability was assessed after 72 h treatment. Briefly, 0.50 μL from a 500× stock of each TPE in DMSO was added directly into each well of the tissue culture plate in a 250 μL final volume of medium and the solution was gently mixed by pipetting.

### 2.5. Cell Viability Assay

Cell viability was evaluated using the MTT colorimetric assay as previously described [20,31]. Briefly, at the end of the treatment with OOPs, cells were incubated for 3 h with 0.5 mg/mL MTT solution. Τhen, the formazan crystals were dissolved in 100 μL DMSO and the optical density of each sample was measured at 570 nm on a microplate reader (Dynatech Laboratories MRX Microplate Reader, Chantilly, VA, USA). Results from two or three independent experiments performed in triplicates were presented either in Tables or in Bar Graphs as mean cell count ± S.E. for each treatment group normalized to the control group [cells treated with 0.2% (*v*/*v*) DMSO].

### 2.6. Evaluation of the Effect of Combinations of OOPs by Modified CDI

The combined effect of the OOPs in the combinations’ treatments was assessed by the coefficient of drug interaction (CDI). CDI = AB/(A × B), where A is the percentage (%) of viable cells after treatment with substance A used alone and Β is the percentage (%) of viable cells after treatment with the 2nd substance used alone. AΒ corresponds to the percentage (%) of viable cells after treatment with the combination of the two compounds. The absorbance ratio of each group was normalized to the value obtained for the control group. Thus, CDI < 1, =1 or >1 indicates drugs with synergistic, additive or antagonistic action, respectively. Drugs with a calculated CDI < 0.7 exert a strong synergistic effect [32,33].

### 2.7. Statistical Analysis

All data were derived from two or three independent experiments performed in triplicates. EC_50_ determination and statistical analyses were performed using the GraphPad Prism v8 (GraphPad Software Inc., San Diego, CA, USA) and Office Excel 365 (Microsoft, Redmond, WA, USA). For the cell-viability assays, the data were normalized to the average absorbance of the control group, which was considered as 100% viability.

Statistical analysis to determine whether OOPs’ anti-proliferative/cytotoxic effect is significant or not was performed by calculating the mean values from the independent experiments and the S.E.s [Excel Formula: SE = STDEV (A1, A2, A3)/SQRT (COUNT (A1, A2, A3). Subsequently, the unpaired, two-tailed t-student test was applied and *p* values were estimated using the GraphPad algorithm.

## 3. Results

### 3.1. Synergistic Effect of OOPs’ Double Combinations on the Cell Numbers/Viability of Cancer and Non-Cancer Cell Lines; Determination of CDI Values

A recently published study by our team reported new affordable methods for the isolation of major secoiridoid derivatives of OOPs and their anti-proliferative and cytotoxic bioactivities in a large array of cancer and non-cancer cell lines, calculating the EC_50_ values and the relative bioactivity for five OOPs for each tested cell line [20]. As a follow-up of this study, synergistic, additive or antagonistic effects of these olive secoiridoids were investigated since they are found in different parts and products of the olive plant in various combinations.

For this purpose, several cancer cell lines were treated with double combinations of OOPs. The cell lines chosen for this analysis where those that showed a lower response to the five OOPs used as single compounds in 72 h treatments in our previous study [20]. Herein, it was chosen to test the OOPs at ½ of the EC_50_ value determined by Papakonstantinou et al. 2022 [20] for each OOP in each tested cell line. Cell viability was measured with the MTT assay.

Several unexpected observations were made concerning the synergistic action of OOPs in double combinations. Although an individual oleomissional appeared to be among the less bioactive OOPs in the treatment of most cancer cells lines with EC_50_ values amongst the highest values (≥34.8 μΜ) [20], in this study it stood out for its synergy with οleuropein aglycone (CDI = 0.3–0.9) in all cell lines tested (Table 1).

**Figure 1:**
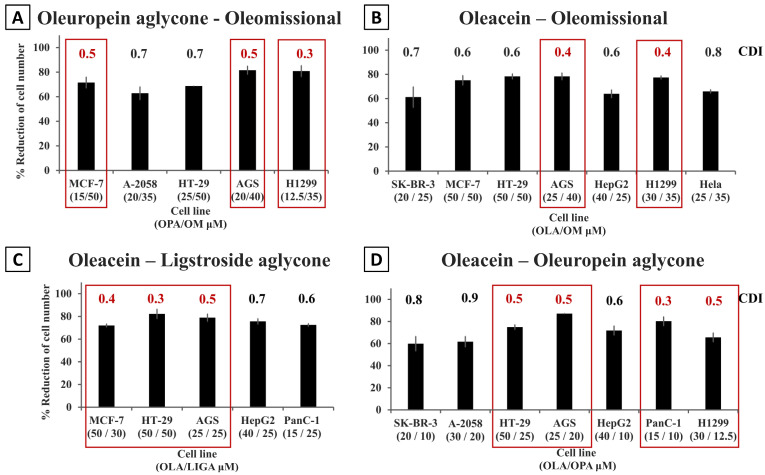
Treatment with double combinations of OOPs that caused a >60% reduction in cell numbers/viability of several cancer cell lines from different tumor origins. The efficacy of four different OOPs (i.e., oleuropein aglycone, ligstroside aglycone, oleacein and oleomissional) when used in four different double combinations is presented as bar graphs. The synergistic action of the four most effective combinations (>60% reduction in cell viability) are shown in (**A**) oleuropein aglycone (OPA) with oleomissional (OM), (**Β**) oleacein (OLA) with oleomissional (OM), (**C**) oleacein (OLA) with ligstroside aglycone (LIGA) and (**D**) oleacein (OLA) with oleuropein aglycone (OPA). Experiments were performed as described in Materials and Methods sections. The results are derived from two independent experiments performed in triplicates. Data are mean cell numbers ± SE in each treatment group normalized to the control group [cells treated only with 0.2% (*v*/*v*) DMSO]. CDI values for each combination were calculated and presented on top of each bar. The most effective combinations with the lowest CDIs (i.e., highest synergistic action) are framed in red.

Double combination treatments with oleomissional and other OOPs resulted in reductions of cell numbers from 44.4 ± 1.9% to 81.6 ± 3.5%, depending on the cell line (Figure 1). The H1299 colon carcinoma, AGS stomach cancer and the MCF-7 breast cancer cell lines were the most sensitive to the oleomissional/oleuropein aglycone double combination (Table 1A, Figure S1). As for the combinational treatment of oleomissional with oleacein, an inhibition of cell viability > 60% tested was observed on seven out of the eleven cell lines, with calculated CDI values indicating synergistic interactions. Interestingly, the synergistic effect was also detected in some cell lines for oleomissional and ligstroside aglycone as well as for oleacein with oleuropein aglycone (Figure 1 and Figure S1).

The AGS, PANC-1 and H1299 cells showed the best response in terms of sensitivity to oleacein combined with either of the two aglycones (reduction of cell numbers ≥ 60% with total OOPs’ concentration ≤ 50 μM) (Figure S1). Oleocanthal, in combination with oleuropein aglycone, also had a good synergistic effect (CDI = 0.4–0.9), a very valuable result given that these two OOPs showed the strongest anti-proliferative/cytotoxic effect when tested alone [lowest EC_50_s in the treatment of most cell lines tested (Tables I and II in Papakonstantinou et al. 2022 [20])]. The most sensitive cell lines to the aforementioned combination were the MCF-7, AGS, PANC-1 and H1299 cell lines (reduction of cell numbers > 68% and calculated CDI ≤ 0.6).

In more detail, from the three breast cancer cell lines tested, the MCF-7 cells were the most sensitive to treatment with most OOP combinations. The oleacein/ligstroside aglycone combination in particular dramatically reduced the MCF-7 cell numbers (i.e., ~70%) with a strong synergistic effect (i.e., CDI = 0.4) (Figure S1). For the MDA-MB 231, double combinations of oleuropein aglycone with oleocanthal, ligstroside aglycone or oleomissional seemed to be the most effective and slightly synergistic (CDI = 0.9; reduction of cell numbers > 40%). The combination of oleuropein aglycone with oleomissional (OPA/OM at 10/25 μM) was most effective on SK-BR-3 cells (CDI = 0.7 and % reduction of cell numbers 57.3 ± 4.5). Double combinations of ligstroside aglycone with oleocanthal or oleacein showed weak synergy (CDI = 0.8), but they were not so effective (reduction of cell numbers < 40%).

Among the skin cancer cell models, the A2058 cell line was the most sensitive to the combination of ligstroside aglycone with oleuropein aglycone [CDI = 0.5; reduction of cell numbers 78.2 ± 2.7% with total [OOPs] = 55 μM]. The best reduction of cell numbers with the lowest total [OOPs] was observed for the combination oleocanthal/oleuropein aglycone (total [OOPs] = 30 μM; 63.8 + 5.1% reduction of cell numbers; CDI = 0.8). Double combinations of ligstroside or oleuropein aglycones with oleomissional also exhibited synergistic effects (CDIs = 0.7; % reduction of cell numbers ≥ 60% using total [OOPs] = 55–70 μΜ) similar to what was observed for the combinations of oleacein with either one of the two aglycones (Table 1A). Oleacein combinations with oleuropein aglycone or oleomissional exerted a relatively good synergistic effect on the SK-MEL-28 cells (CDI = 0.6–0.7; reduction of cell numbers ≥ 50%; total [OOPs] = 22–35 μM).

From the colon and gastric carcinoma cell models, the AGS stomach cancer cell line appeared to be the most sensitive to double OOP combination treatments. More specifically, the AGS cells were very sensitive to combinations of οleomissional with oleacein or ligstroside aglycone, showing the strongest synergistic effect (CDI = 0.4; reduction of cell numbers > 70–80% at total [OOPs] = 60–65 μM). However, at lower total [OOPs], the best effect on the AGS cells was observed for the combination of οleocanthal with either οleuropein aglycone or ligstroside aglycone (i.e., reduction of cell numbers 67.2 ± 0.9–78.9 ± 2.4%; CDIs = 0.5–0.6; [OOPs] = 30–35 μM). Although the HT-29 colon cancer cells appeared to be more resistant than the AGS to the majority of the OOP combinations tested in the study, the oleacein/ligstroside aglycone combination dramatically reduced the cell numbers (82.2 ± 4.3%; [OOPs] = 100 μM) in this cell line, with a very strong synergistic effect (CDI = 0.3) also observed for the MCF-7 breast cancer cells.

The HepG2 liver cancer cell line responded well (i.e., reduction in cell numbers 44.4 ± 1.9–80.2 ± 3.7%; CDI = 0.6–0.8; [OOPs] = 30–65 μΜ) to the treatment by most of the OOP double combinations (Table 1A). The oleocanthal/ligstroside aglycone combination and those of oleacein with either one of the two aglycones (ligstroside or oleuropein) had the greatest effect on reducing cell numbers (i.e., 71.8 ± 4.3–80.2 ± 3.7%) at total [OOPs] from 35–50 μM. Moreover, the combination of the two aglycones showed a strong anti-proliferative/cytotoxic effect on the liver cell line (inhibition 75.3 ± 4.8%, CDI = 0.7), using low total [OOP] (≤25 μΜ).

The PANC-1 pancreatic cancer cell line was the most sensitive of all cancer cells studied herein to all the double combinations of OOPs used in the treatments (i.e., percentages of cell reduction 43.8 ± 1.4–81.8 ± 2.9% for all combinations of total [OOPs] = 18–42.5 μM; (Table 1A); CDI = 0.3–0.8). Six out of the ten combinations tested presented strong synergistic effects (CDI = 0.3–0.6) while the other four showed moderate synergistic effects (CDIs = 0.7–0.8). The H1299 lung cancer cells were also very sensitive to the combinatorial treatment, with the best highlighted combinations being those of oleuropein aglycone/oleomissional, oleocanthal/ligstroside aglycone and oleacein/oleomissional (reduction of cell number 77.4 ± 1.5–80.9 ± 4.6% with [OOPs] = 47.5–65 μM; CDIs = 0.3–0.4). Finally, the Hela cervical carcinoma cells seemed to respond moderately well to OOP double combinations (reduction of cell numbers 55–78% at [OOPs] = 35–60 μM), with the best combination treatments being the oleocanthal with either of the two aglycones (i.e., ligstroside or oleuropein) and the oleomissional with oleocanthal, ligstroside aglycone or oleacein (CDIs = 0.7–0.8)

Overall, several double OOP combinations at ½ EC_50_ concentrations of each OOP used in the combinatοrial treatments were strongly effective at inhibiting proliferation or viability in all cancer cell lines tested. The AGS, PANC-1 and H1299 were found to be the most sensitive cell lines in most double combinations applied (Figure 1, Figures S1 and S2 and Table 1). Interestingly, a variability on the effects of the double combinations was observed for each cell line as for the estimation of the EC_50_ values of each single OOP tested. The compilation of the results presented in Table 1 highlights that all OOPs present synergistic effects in various double combinations in almost all cell lines. Interestingly, in the breast cancer cell lines the double combinations of oleacein with oleocanthal, ligstroside aglycone, oleuropein aglycone or oleomissional highlighted a slightly antagonistic (CDI = 1.1) or simply additive effect (CDI = 1) between these OOPs. The only other cell line for which an antagonistic effect was observed (CDI = 1.1) for a double OOP combination (i.e., oleacein/ligstroside aglycone) was the HeLa cervical epithelial cells.

The cytotoxicity against non-tumorigenic cell lines is an assessment of high importance for the evaluation of the potential value of a compound in cancer treatment protocols. The selectivity index is a ratio that determines the window between cytotoxicity and anticancer activity. (SI: ratio of the % inhibition for non-tumorigenic cells/% inhibition for cancer cells) [34]. This parameter was investigated in the double combinations of OOPs which showed the maximum synergistic effect in the three breast cancer or the two melanoma cell lines. Their anti-proliferative/cytotoxic action was evaluated under similar conditions of treatment (i.e., 72 h) in the human non-cancerous breast cell line MCF 10A and the HaCaT non-cancer skin cells, and the selectivity indices were calculated. As shown in Table 1B, the MCF 10A cells were very sensitive to all double combinations while in the HaCaT cells the combination of the two aglycones showed the highest selectivity index (SI = 1.8, Table 2) of all the combinations tested. The least reduction on cell numbers for the HaCaT cell line was observed for the oleocanthal/oleacein (42.0 ± 6.0%) and oleacein/oleomissional (42.5 ± 2.0) combinations at total [OOPs] = 40–65 μM. The melanoma cell lines were slightly more sensitive to these combinations. Treatment of the SK-MEL-28 and A2058 cells with the combination of oleocanthal and oleacein reduced cell numbers to 43.2 ± 2.2%–55.5 ± 4.3%, respectively, when the total [OOPs] used were at 20–35 μΜ. Therefore, double combinations of oleocanthal/oleacein and oleacein/oleomissional would be less harmful on the HaCaT immortalized keratinocyte cell line if used at total concentrations of 20–35 μΜ (Table 1B).

The selectivity indices of the combinations for the cell lines (cancer and non-tumorigenic transformed cells) originating from the same tissue summarized in Table 2 ranged between 1–1.8. The SIs for treatments with all double combinations of OOPs were ≥1 and in most cases > 1.2 for cells of skin origin (Table 2), an interesting and promising result for future translational applications. Most promising in terms of selectivity was the double combination of ligstroside aglycone with oleuropein aglycone. This combination was almost two-fold less toxic for the non-cancer cells tested.

### 3.2. Anti-Proliferative/Cytotoxic Effect of TPEs on Several Cancer and Non-Tumor Derived Cell Lines

Τhe observations on the synergistic, additive or in a few cases antagonistic effects of certain OOP double combinations paved the way for proceeding in testing samples of total phenolic extracts isolated from olive oil produced from olives of different cultivars. Two of the TPEs used had higher contents in oleocanthal (the most bioactive OOP of all tested) and contained all the other OOPs for which the EC_50_ values were determined in our previous study [20], while the third TPE was devoid of oleocanthal and oleacein (Table S3). Moreover, TPE I contained oleokoronal [18] and tyrosol but lacked oleomissional, while TPE II contained oleokoronal and oleomissional but lacked tyrosol. TPE III contained oleokoronal, oleomissional and tyrosol (Table S3). TPEs I and II had very similar concentration of oleocanthal (i.e., 53.6 and 50 mM respectively), but exhibited differences in the concentrations of ligstroside aglycone, oleomissional, oleokoronal and tyrosol (Table S3). TPE III, besides lacking oleocanthal and oleacein, had higher contents than the other two TPEs in ligstroside aglycone, oleuropein aglycone, oleokoronal and tyrosol (Table S3).

For the evaluation of the anti-proliferative/cytotoxic efficacy of the three TPEs, all the breast cancer (i.e., MDA-ΜΒ 231, SK-BR-3 and MCF-7) and melanoma (SK-MEL-28, A2058) cell lines available for this study were selected for testing, as well as the cell lines from each other cancer type studied herein which were less sensitive to the treatment by the individual OOPs contained in the TPEs (except tyrosol and oleokoronal, which were not tested as individual compounds in our previous study [20]). TPE III was tested only in six out of the eleven cell lines evaluated in this study. The reason was that in all six cases, TPE III showed lower efficacy than the other two TPEs containing oleocanthal, the OOP with the highest efficacy. The same protocol was followed for the treatments with the double combinations of isolated OOPs. The EC_50_ values of the two TPEs for each cell line tested are presented in Table 3.

**Figure 2:**
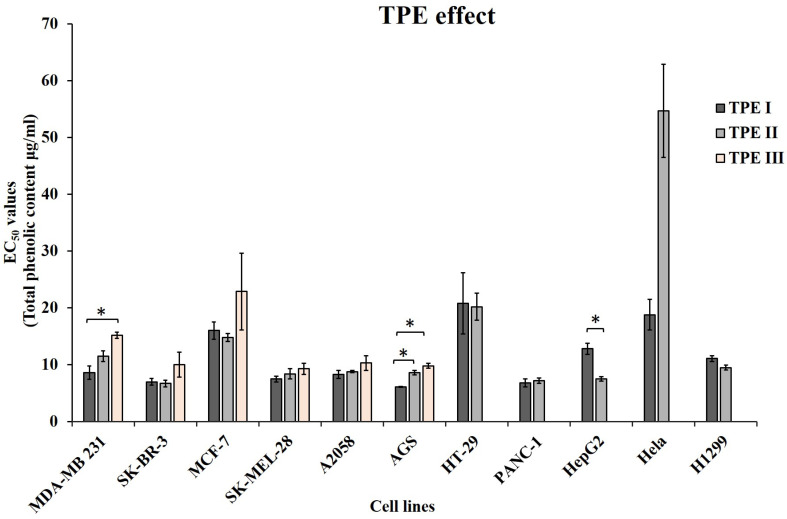
Olive oil TPEs reduce cell viability of eleven cancer cell lines from different tumor origins. The effect of different concentrations of three TPEs with different compositions of OOPs on a panel of eleven cancer human cell lines from eight different cancer types was evaluated as described in Materials and Methods sections. Bar graphs represent the mean EC_50_ values ± SE. Results are means ± SE from two independent experiments conducted in triplicates (total no. of cells ≥ 1000). * *p* < 0.05.

As expected from each different composition of TPEs in OOPs, the levels of sensitivity to the treatments differed in the different cell lines (Table 3 and Figure 2). For example, TPE I reduced the number of the MDA-MB 231 cells by 50% at a concentration of total phenolic content equal to 8.6 ± 1.2 μg/mL, TPE II at 11.5 ± 0.9 μg/mL and TPE III at 15.2 ± 0.5 μg/mL. Concerning the HepG2 cells, the EC_50_ for TPE I was 12.8 ± 1.0 μg/mL, while for TPE II it was 7.5 ± 0.4 μg/mL. On the contrary, the HepG2 cells were more sensitive to treatment with TPE II than with TPE I (EC_50_ values 7.5 ± 0.4 and 12.8 ± 1.0 μg/mL, respectively). As shown in Figure 2, TPE I had stronger anti-proliferative/cytotoxic activity when compared to TPE II in six out of the eleven cancer cell lines tested (EC_50s_ of TPE I < EC_50_s of TPE II). With respect to the cells treated with all three TPEs, it seems that in all cases they respond less to the treatment with TPE III as compared to treatment with the other two TPEs. The difference in the bioactivity of TPEs I and II was significant (*p* ≤ 0.05) in the AGS and HepG2 cells, while between the TPEs Ι and ΙΙΙ a significant difference (*p* ≤ 0.05) was observed in the MDA-MB 231 and AGS cell lines.

It is possible that the higher content in oleocanthal and oleacein, as well as the presence of tyrosol in TPE I (Table S3), might have contributed to its stronger anti-proliferative/cytotoxic bioactivity (ΕC_50_ TPE I < ΕC_50_ TPE ΙΙ; Table 3). Amongst the few exceptions where the opposite was observed was the case of the HepG2 hepatocellular carcinoma cells, which were more sensitive to TPE II as compared to TPE Ι. Interestingly, TPE ΙΙ contained ligstroside aglycone and oleokoronal, the presence of which may contribute to the higher efficacy of TPE II in these cells. On the contrary, the absence of oleocanthal and oleacein from TPE III seemed to reduce its efficacy (ΕC_50_ of TPE ΙΙΙ > ΕC_50_ of TPEs Ι and ΙΙ) despite its higher content in the other two of the most bioactive OOPs (i.e., ligstroside aglycone and oleuropein aglycone). The EC_50_ value of oleocanthal, which is the most abundant OOP in the two TPEs (44.6% in TPE I and 33.9% in TPE II), was lower than all the EC_50_ values of TPEs I and II when used alone as a single compound (2.8–13.6 μg/mL). This could be explained by the antagonistic effect observed amongst oleocanthal and some of the OOPs in the double OOP combination experiments described in Section 3.1 (CDIs in Table 2).

When the EC_50_ values of the TPEs were compared with those of each individual phenol for the same cell line, it was observed that in about 53–64% of cases the TPEs were more effective—even by more than two-fold—or in some cases they had at least equal efficacy. The case in which the exact opposite was observed mainly concerned oleocanthal, and in some cases the aglycone of oleuropein. It is interesting to highlight that the EC_50_ value of oleocanthal as a single phenol [20] was lower than the EC_50_ values of TPEs I and II in which it is the most abundant phenol (44.6% in TPE I and 33.9% in TPE II) for all cell lines. The sole exception was the EC_50_ value of TPE II for HepG2 cells. A similar pattern was observed in the Hela cell line for all phenols as compared to TPEs I and II and in the MDA-MB 231 cell line for TPE III. These results confirm the existence of the antagonistic action observed between some of phenols in the experiments described above with the double OOP combinations (Table 1).

In summary, different combinations of multiple phenols in the olive oil TPEs from different olive cultivars had different effects on the proliferation/viability of different cancer cell lines ex vivo. However, in almost all cases incubation with oleocanthal as a single substance showed the strongest effect in reducing cell number/viability. Moreover, of all the cell lines tested HeLa appeared to be the least sensitive to incubation with TPEs I and II, while the effect of each individual OOP was stronger on these cells as compared to that of the TPEs, an interesting observation which merits further investigation regarding the mechanism of action of these OOPs in this cell type.

Tyrosol in TPE I seemed to act synergistically with other OOPs in the reduction of cell numbers in nine out of the fourteen cell lines tested (EC_50_s of TPE I < EC_50_s of TPE II). In two cell lines (MCF-7 and HepG2) TPE II was more effective, while in four others (SK-BR-3 and HT-29, PANC-1 and H1299) the EC_50_s of the two TPEs were very close (Table 3A). In the HaCaT non-cancer skin cells and in the HELA cervix carcinoma, the presence of tyrosol in TPE I seemed to significantly increase its efficacy. However, TPE I is also richer by 10.7% in oleocanthal and by 5.3% in oleacein. TPE II is richer in ligstroside aglycone by 11.5% and in oleokoronal by 11.3% compared to TPE I and contains 3.3% oleomissional not present in TPE I. The higher efficacy of TPE II in HepG2 cells highlights a potential role for oleokoronal in the reduction of cell numbers in this cell line. It suffices to say that HepG2 cells appeared to respond less in the treatment with oleocanthal [20], something that was confirmed by the lower efficacy of TPE I to reduce cell numbers in this cell line.

The olive oil TPEs I and II were also studied for their anti-proliferative/cytotoxic activity in the non-cancer HaCaT and NHDF skin cell lines and in the MCF 10A cells from breast tissue. Cell viability was assessed using the MTT assay after 72 h treatment with the TPEs. The MCF 10A cell line proved to be the most sensitive to the two TPEs tested (TPE I & II) as observed for the double OOP combinations, while the skin cell lines appeared to have a similar response to the melanoma cell lines. As for the MCF 10A cells, it was observed that they were more sensitive to treatment with TPE I than with TPE II (EC_50_ = 5.2 ± 0.5 μg/mL and 12.8 ± 0.8 μg/mL, respectively (Table 3B)). In addition, they were more sensitive to both phenolic TPEs than two of the three breast cancer lines (i.e., the MDA-MB-231 and MCF-7 cells). An exception was the SK-BR-3 cells, which proved to be more sensitive than the non-neoplastic MCF 10A cells to treatment with TPE II, with an EC_50_ value half of that observed for the non-neoplastic MCF 10A cells. Moreover, TPE I was more active than TPE II in all the breast cancer cells. As for the HaCaT and NHDF skin cell lines, the calculated EC_50_ values for the two TPEs were higher than those calculated for the MCF 10A and all the breast cancer cell lines. Finally, comparing the EC_50_ values of the non-cancerous skin lines with the corresponding ones of the melanoma cell lines was observed to produce a similar response to the treatment with TPE I, while the cancer cells responded better to TPE II.

Calculations of the selectivity indices (SIs) for the cell lines of breast and skin origin showed that TPE II was less toxic (SI = 1.7–2.1) for non-cancer cell lines of both skin and breast origin (Table 4). The highest SI values were observed for the breast cell line SK-BR-3 and for the melanoma cell lines A2058 and SK-MEL-28 (SI = 1.7–2.1).

Moreover, when the EC_50s_ of the two TPEs were compared to the EC_50_ values of every single OOP component tested on the same cell line, it was observed that in 64% of the cases the TPEs were more effective, even by more than two-fold, or were at least of equal efficacy (Table 5). Only oleocanthal was found to be more effective than the three TPEs in all the tested cell lines except for HepG2, in which the TPE II had a lower EC_50_ (ratio 0.6). Moreover, in the HepG2 cells oleuropein aglycone had almost equal efficacy to TPE II (ratio 1.1) and a two-fold higher efficacy than TPE I (ratio 1.9) (Table 5).

In summary, olive oil TPEs of different composition in OOPs have different efficacies in inhibiting proliferation/viability of different cancer as well non-cancer cell lines. However, in all cases except for the HepG2 hepatoma cells, treatment with oleocanthal as a single compound showed the strongest effect in reducing cell numbers/viability. Interestingly, the HeLa cells seemed to be the least sensitive cell line to the two TPEs tested on them (i.e., TPE I and II), responding better in all the OOPs tested as individual compounds (Table 5).

## 4. Discussion

Pre-clinical studies on the anticancer effect of polyphenols from different plants have demonstrated that combinational treatment of two or three polyphenols inhibited cancer growth more effectively than a single compound treatment. The synergistic action between multi-nutrients can modulate natural products’ bioavailability and affect in a pleiotropic way multiple metabolic pathways involved in carcinogenesis simultaneously [27].

The study presented herein demonstrates strong synergistic effects in most of the possible double combinations of five olive oil secoiridoids in treatments of eleven cancer cell lines from eight tissue origins. The existence of synergy between the OOPs under study is very important as it allows the reduction of the active concentrations for each substance and possibly the reduction of its toxic effect on normal cells in protocols for in vivo study of their anticancer efficacy. In addition, evaluation of the anti-proliferative/cytotoxic activity of olive oil TPEs, as compared to individual phenols, is a field of great interest for anticancer therapy, given that the microenvironment of TPEs could act as a means of easier uptake of OOPs by the cells, modify their bioavailability and enhance the efficacy of individual phenols with complementary or synergistic interactions.

Some very interesting conclusions emerged in this study regarding the synergistic action of OOPs in double combinations. Specifically, two of the least bioactive OOPs were highlighted for their synergistic action with the other four OOPs tested. Oleomissional, when combined with each of the other four OOPs at concentrations of ½ the EC_50_ value for each substance, inhibited cell viability in percentages ranging from 35% to 80%, depending on the cell line and the OOP used in the combination. In fact, the CDI coefficient was less than one—in the majority of cases—indicating synergistic interactions between the tested OOPs. The combination of oleomissional which stood out for its synergy was that with oleacein (also an OOP with weak efficacy). Treatment with oleomissional and oleacein inhibited cell viability by up to 80% in some cases. It is interesting to note that this particular OOP combination caused a greater than 60% reduction in cell viability in seven out of the eleven cancer cell lines tested, with the AGS stomach cancer and H1299 lung cancer cell lines proven to be the most sensitive. In addition, good synergistic action was observed between oleacein and the two aglycones. In particular, incubation with the combination of oleacein and ligstroside aglycone appeared to have stronger efficacy in the breast, colon, stomach and pancreas cell lines (inhibition > 70% at concentrations ≤ 25 µM), surpassing the efficacy of the individual OOPs.

The most bioactive double OOP combinations were tested in non-cancer cell lines. The MCF 10A breast cell line proved to be very sensitive to all dual OOP combinations, while the non-cancerous skin cell line HaCaT was relatively less sensitive to the combination therapy. The highest selectivity index was obtained for the combination of the two aglycones (oleuropein and ligstroside) in the skin cell lines, while the melanoma cell lines were slightly more sensitive to the combinations of oleocanthal—oleacein and oleacein—oleomissional as compared to the non-cancerous HaCaT cells.

In general, the AGS, PANC-1 and H1299 cell lines were found to be the most sensitive lines to most of the double combinations applied, while phenols with lower efficacy, when used in the treatments as single compounds (i.e., oleacein, oleomissional), showed a strong synergistic effect with all the rest. Finally, in most cases there was synergy between the OOPs studied, with the exception of some of the combinations with oleacein and oleocanthal in breast cell lines, where either additive or antagonistic effects were observed.

Evaluation of the bioactivity of the double OOP combinations was followed by the study of the anti-proliferative/cytotoxic bioactivity of olive oil TPEs with different contents of the five OOPs under study and of other OOPs. As expected, differences in the composition of each TPE in terms of bioactive phenols resulted in differences in their efficacy to reduce cell numbers in the cell lines incubated with them. In particular, TPE I, which contained more oleocanthal and oleacein than TPE II (similar OOP composition with small differences in percentages), was more active in most cell lines tested, suggesting that these two OOPs contribute to the higher efficacy of TPE I to reduce cancer cell viability. It is also possible that this difference is due to the presence of tyrosol in TPE I. Tyrosol is a phenolic compound of olive oil mostly known for its antioxidant and anti-inflammatory properties and for the ability to act as radical chelator. Chronic inflammation and alterations of normal cellular redox status are two main factors that promote neoplastic transformation, so tyrosol may act as an anticancer or chemopreventive agent in different human malignancies [3]. An exception to the higher efficacy of TPE I was observed in the case of HepG2 liver cells, which were found to be more sensitive to TPE II treatment. This may be due to the increased content of ligstroside aglycone and oleokoronal in TPE II. Previous studies with liver cancer cells on the bioactivity of olive oil TPEs rich in oleocanthal and ligstroside aglycone align with the aforementioned result [35]. Moreover, the increased efficacy of TPE II in this liver cell line may also be due to the existence of oleomissional, which could enhance the activity of the other phenols. As shown in the results of the double combinations (Table 1 and Figure 1), oleomissional showed a strong synergistic effect with the rest of the bioactive phenols.

TPE III, which lacked the oleocanthal and oleacein present in the other two TPEs, appeared to have equal or slightly lower efficacy in all cell lines tested. In fact, the difference in the bioactivity of TPE III as compared to TPE I appeared to be significant only against the breast cell line MDA-MB 231 and the stomach AGS cells. This could be due to the presence of oleocanthal, which is the most bioactive molecule of all the tested OOPs in these cell lines [20].

Overall, in evaluating the results reported in this study regarding the anti-proliferative/cytotoxic activity of the three TPEs for each cancer type tested and comparing them with results already reported by other researchers, a number of interesting observations emerged. Regarding the MCF-7 breast cancer cells, the study by Reboredo-Rodríguez et al. 2018 showed that an olive oil extract with higher levels of the two aglycones (ligstroside and oleuropein) than of oleocanthal and oleacein inhibited cell viability by 50% at a concentration of about 500 µg/mL in 48 or 72 h treatments [36]. This concentration is >25 fold higher than the EC_50_ values calculated for the three olive oil TPEs in the present study for the specific cell line (i.e., 14.8–22.9 µg/mL). An explanation for this quantitative difference between the results reported herein and those by Reboredo et al. 2018 [36] may be due to the instability of some OOPs in the culture medium. In that study, the dried extract had been directly dissolved in the cell culture medium and then the mixture was added to the cells for the treatment. As we have recently shown [20], any unintentional delay in this step may lead to partial deactivation of some OOPs of the extract, leading to significantly weaker activity. Moreover, in that study the most critical secoiridoids had been tentatively quantified chromatographically without pure standards, in contrast to the current study, which is based on qNMR. The accuracy of the quantitation method by which the concentration and the ratio of OOPs in the TPEs is determined plays a crucial role in the comparison of the results.

In this study, the melanoma cell lines SK-MEL-28 and A2058 showed a similar response to all three TPEs tested. Previous studies [37] have also reported that olive oil TPEs with differences in their compositions of OOPs (i.e., oleocanthal:oleacein ratio 70%: 26% (TPE1) and 37%: 60% oleacein (TPE2)), and with low content of other OOPs, reduced the viability and migration of non-melanoma cancer cells and showed similar EC_50_s in the A431 epidermal cancer cells after 72 h incubation (i.e., TPE1 EC_50_: 37.4 ± 1.1 and TPE2 EC_50_: 46 ± 1.1 µg/mL) [37]. However, the EC_50_ values calculated in that study were four- to five-fold higher than the EC_50_s reported in this study for the melanoma cell lines A2058 and SK-MEL-28 for TPE I and TPE II (i.e., 7.5–10.3 μg/mL). This may be due to the difference in the cell lines tested, which, although originating from the same tissue, are derived from different kind of cancers. Μoreover, as observed for the breast cancer cell lines, a significant difference in the efficacy of TPEs may be due to the different concentrations of the secoiridoid polyphenols in the three different extracts and especially the absence of oleocanthal in the TPE III as well as to the method by which the OOP concentrations in the TPEs were determined.

Furthermore, the results of the present study showed that the HT-29 colon cancer cells responded to higher concentrations of all three olive oil TPEs (EC_50_ ~ 20 µg/mL) compared to the other cancer cell lines tested. Nevertheless, other ex vivo and in vivo studies with olive oil TPEs on colon cancer have shown inhibition of certain critical steps in the development of carcinogenesis and in the invasive and migratory capacity of cancer cells [38,39]. An additional study by Pampaloni et al. 2014 demonstrated that TPEs inhibited cell proliferation in colon cancer cells with activation of receptors, acting similarly to 17β-estradiol [40].

Comparison of the TPEs’ EC_50_ values with the EC_50_s of individual OOPs in treatments of the same cell line showed that oleocanthal has the strongest efficacy in reducing cell number/viability. In most cases, the rest of the OOPs were surpassed by the bioactivity of the TPEs. An exception was the HeLa cells, which appeared to be the least sensitive to incubation with the two TPEs tested (I and II), while the effect of each individual OOP on these cells was stronger compared to that of the TPEs. Finally, an evaluation of the selectivity of TPEs I and II on breast and skin cell lines showed that TPE II had a higher selectivity index (SI = 1.1–2.1) as compared to TPE I (SI = 0.3–1.5). However, some individual OOPs have been shown to have higher Sis [20].

## 5. Conclusions

The results presented herein demonstrate synergistic interactions between several OOPs in double combinations and in the context of olive oil TPEs, enhancing their anti-proliferative/cytotoxic efficacy in treatments of several cancer cell lines. As the existing chemotherapeutic approaches present problems of side effects in normal tissues and resistance development in cancer cells, there is a constant need for more and alternative anticancer agents and new strategies in current cancer treatments. Combinations of anticancer drugs with plant polyphenols in chemotherapeutic regimens hold promising value. Certain OOPs can be added to the list of promising polyphenols for this approach. Combinations of OOPs or olive oil TPEs enriched with the most bioactive OOPs represent good candidates as well. The results of the present study form a base for further in vivo studies in cancer animal models and clinical trials investigating the chemopreventive and anticancer value of OOPs and TPEs.

## Figures and Tables

**Table 1:** Evaluation of the synergistic anti-proliferative/cytotoxic activity of OOPs in cancer and non-cancer cell lines. (**A**) All the breast cancer and melanoma cell lines and cell lines from other cancer types studied in Papakonstantinou et al. [20] which were available for this study and found to be less sensitive to the activity of individual OOPs. (**B**) Non-cancer cell lines from breast (i.e., MCF 10A) and skin (i.e., HaCaT). Treatments with double OOP combinations were performed as described in Materials and Methods. Oleocanthal: OLC; oleacein: OLA; ligstroside aglycone: LIGA; oleuropein aglycone: OPA; oleomissional: OM.

**A.**					
**Cell Origin**	**Cancer Cell Line**	**Combination**	**Concentration** **(OOP1/OOP2 μΜ)**	**% Inhibition of Viability**	**CDI**
Human breast cancer cell lines	MDA-MB 231	OLC/OLA	5/15	20.3 ± 2.4	1.1
		OLC/LIGA	5/15	30.4 ± 4.2	0.8
		OLC/OPA	5/15	42.4 ± 1.7	0.9
		OLC/OM	5/35	42.9 ± 2.4	1.0
		OLA/LIGA	15/15	36.0 ± 3.2	0.8
		OLA/OPA	15/15	35.2 ± 4.7	1.1
		OLA/OM	15/35	41.9 ± 5.4	1.1
		LIGA/OPA	15/15	39.4 ± 5.4	0.9
		LIGA/OM	15/35	35.5 ± 2.5	1.0
		OPA/OM	15/35	56.9 ± 4.8	0.9
	SK-BR-3	OLC/OLA	6/20	44.8 ± 1.2	1.1
		OLC/LIGA	6/10	38.3 ± 1.5	0.9
		OLC/OPA	6/10	53.6 ± 3.9	0.9
		OLC/OM	6/25	48.7 ± 1.3	0.9
		OLA/LIGA	20/10	38.1 ± 4.4	1.0
		**OLA/OPA**	**20/10**	**59.9 ± 6.7**	**0.8**
		**OLA/OM**	**20/25**	**61.2 ± 8.6**	**0.7**
		LIGA/OPA	10/10	46.5 ± 0.9	0.8
		LIGA/OM	10/25	38.2 ± 6.1	0.9
		OPA/OM	10/25	57.3 ± 4.5	0.7
	MCF-7	OLC/OLA	10/50	51.5 ± 4.1	1.0
		OLC/LIGA	10/30	46.5 ± 6.5	0.7
		**OLC/OPA**	**10/15**	**69.1 ± 0.8**	**0.5**
		OLC/OM	10/50	54.2 ± 3.0	1.0
		**OLA/LIGA**	**50/30**	**72.0 ± 1.5**	**0.4**
		OLA/OPA	50/15	43.2 ± 5.0	1.0
		**OLA/OM**	**50/50**	**75.1 ± 4.1**	**0.6**
		LIGA/OPA	30/15	48.9 ± 7.4	0.6
		**LIGA/OM**	**30/50**	**69.8 ± 2.8**	**0.5**
		**OPA/OM**	**15/50**	**71.5 ± 4.6**	**0.5**
Skin melanoma cell lines	SK-MEL-28	OLC/OLA	5/15	43.2 ± 2.2	0.8
		OLC/LIGA	5/10	25.5 ± 3.5	0.8
		OLC/OPA	5/8	35.7 ± 2.4	0.8
		OLC/OM	5/20	37.4 ± 3.6	0.8
		OLA/LIGA	15/10	32.7 ± 3.5	0.8
		OLA/OPA	15/8	54.0 ± 0.9	0.6
		OLA/OM	15/20	50.7 ± 1.3	0.7
		LIGA/OPA	10/8	29.9 ± 3.1	0.8
		LIGA/OM	10/20	21.9 ± 4.8	0.8
		**OPA/OM**	**8/20**	**50.5 ± 2.3**	**0.6**
	A2058	OLC/OLA	10/30	55.5 ± 4.3	1.0
		OLC/LIGA	10/35	58.2 ± 4.3	0.8
		**OLC/OPA**	**10/20**	**63.8 ± 5.1**	**0.8**
		OLC/OM	10/35	48.2 ± 2.9	0.9
		OLA/LIGA	30/35	59.4 ± 4.7	0.8
		**OLA/OPA**	**30/20**	**61.7 ± 4.9**	**0.9**
		OLA/OM	30/35	53.3 ± 4.7	0.9
		**LIGA/OPA**	**35/20**	**78.2 ± 2.7**	**0.5**
		LIGA/OM	35/35	59.2 ± 2.7	0.7
		**OPA/OM**	**20/35**	**62.8 ± 5.4**	**0.7**
Colon and gastric cancer cell lines	HT-29	OLC/OLA	15/50	47.7 ± 0.8	0.9
		OLC/LIGA	15/50	55.9 ± 5.1	0.6
		OLC/OPA	15/25	49.2 ± 3.7	0.7
		**OLC/OM**	**15/50**	**60.8 ± 3.8**	**0.7**
		**OLA/LIGA**	**50/50**	**82.2 ± 4.3**	**0.3**
		**OLA/OPA**	**50/25**	**74.9 ± 2.3**	**0.5**
		**OLA/OM**	**50/50**	**78.3 ± 2.3**	**0.6**
		**LIGA/OPA**	**50/25**	**71.4 ± 0.2**	**0.5**
		**LIGA/OM**	**50/50**	**71.2 ± 2.3**	**0.6**
		**OPA/OM**	**25/50**	**68.7 ± 0.1**	**0.7**
	AGS	**OLC/OLA**	**10/25**	**64.3 ± 3.4**	**0.8**
		**OLC/LIGA**	**10/25**	**67.2 ± 0.9**	**0.5**
		**OLC/OPA**	**10/20**	**78.9 ± 2.4**	**0.6**
		**OLC/OM**	**10/40**	**54.9 ± 2.3**	**0.7**
		**OLA/LIGA**	**25/25**	**78.9 ± 3.4**	**0.5**
		**OLA/OPA**	**25/20**	**87.1 ± 0.3**	**0.5**
		**OLA/OM**	**25/40**	**78.3 ± 3.0**	**0.4**
		**LIGA/OPA**	**25/20**	**83.3 ± 1.3**	**0.5**
		**LIGA/OM**	**25/40**	**71.3 ± 4.6**	**0.4**
		**OPA/OM**	**20/40**	**81.6 ± 3.5**	**0.5**
Liver cancer cell line	HepG2	**OLC/OLA**	**20/40**	**67.8 ± 3.8**	**0.8**
		**OLC/LIGA**	**20/25**	**80.2 ± 3.7**	**0.6**
		**OLC/OPA**	**20/10**	**62.8 ± 3.9**	**0.8**
		OLC/OM	20/25	57.5 ± 2.4	0.7
		**OLA/LIGA**	**40/25**	**75.6 ± 2.6**	**0.7**
		**OLA/OPA**	**40/10**	**71.8 ± 4.3**	**0.6**
		**OLA/OM**	**40/25**	**63.9 ± 3.4**	**0.6**
		**LIGA/OPA**	**25/10**	**75.3 ± 4.8**	**0.7**
		**LIGA/OM**	**25/25**	**62.3 ± 0.3**	**0.7**
		OPA/OM	10/25	44.4 ± 1.9	0.8
Pancreas cancer cell line	PANC-1	**OLC/OLA**	**8/15**	**67.4 ± 3.8**	**0.5**
		**OLC/LIGA**	**8/25**	**81.8 ± 2.9**	**0.4**
		**OLC/OPA**	**8/10**	**71.6 ± 1.6**	**0.4**
		OLC/OM	8/17.5	59.2 ± 2.7	0.7
		**OLA/LIGA**	**15/25**	**72.5 ± 1.3**	**0.6**
		**OLA/OPA**	**15/10**	**80.2 ± 4.2**	**0.3**
		OLA/OM	15/17.5	43.8 ± 1.4	0.8
		**LIGA/OPA**	**25/10**	**77.6 ± 0.6**	**0.5**
		**LIGA/OM**	**25/17.5**	**68.5 ± 5.0**	**0.7**
		OPA/OM	10/17.5	48.7 ± 2.5	0.7
Lung cancer cell line	H1299	**OLC/OLA**	**10/30**	**63.5 ± 4.1**	**0.6**
		**OLC/LIGA**	**10/40**	**77.9 ± 1.7**	**0.4**
		**OLC/OPA**	**10/12.5**	**68.6 ± 4.5**	**0.5**
		**OLC/OM**	**10/35**	**60.4 ± 3.5**	**0.8**
		OLA/LIGA	30/40	56.3 ± 4.8	0.7
		**OLA/OPA**	**30/12.5**	**65.6 ± 4.2**	**0.5**
		**OLA/OM**	**30/35**	**77.4 ± 1.5**	**0.4**
		**LIGA/OPA**	**40/12.5**	**64.6 ± 4.1**	**0.6**
		**LIGA/OM**	**40/35**	**77.9 ± 4.1**	**0.4**
		**OPA/OM**	**12.5/35**	**80.9 ± 4.6**	**0.3**
Cervix cancer cell line	Hela	OLC/OLA	20/25	73.2 ± 1.3	0.8
		**OLC/LIGA**	**20/25**	**76.2 ± 2.5**	**0.7**
		**OLC/OPA**	**20/15**	**62.0 ± 3.8**	**0.8**
		**OLC/OM**	**20/35**	**78.2 ± 1.9**	**0.7**
		OLA/LIGA	25/25	49.1 ± 5.7	1.1
		OLA/OPA	25/15	39.4 ± 1.6	0.9
		**OLA/OM**	**25/35**	**65.9 ± 1.6**	**0.8**
		LIGA/OPA	25/15	54.9 ± 3.9	0.7
		**LIGA/OM**	**25/35**	**60.3 ± 4.2**	**0.8**
		**OPA/OM**	**15/35**	**55.0 ± 0.1**	**0.7**
**B.**					
**Cell Origin**	**Cell Line**	**Combination**	**Concentration** **(OOP1/OOP2 μΜ)**	**% Inhibition of Viability**	**CDI**
Spontaneously transformed aneuploidy immortal keratinocytes	HaCaT	*OLC/OLA*	*10/30*	*42.0 ± 6.0*	*0.8*
		OLC/LIGA	10/35	58.6 ± 3.7	0.7
		**OLC/OPA**	**10/20**	**51.9 ± 3.3**	**0.9**
		OLC/OM	10/35	50.3 ± 9.2	0.7
		**OLA/LIGA**	30/35	53.7 ± 2.8	0.6
		**OLA/OPA**	30/20	**62.0** ± 7.4	**0.5**
		**OLA/OM**	30/35	42.5 ± 2.0	0.6
		**LIGA/OPA**	35/20	**60.9** ± 8.5	**0.6**
		LIGA/OM	35/35	55.4 ± 9.4	0.5
		OPA/OM	20/35	56.6 ± 3.9	0.6
Human non-tumorigenic epithelial cell	MCF 10A	**OLC/OPA**	**5/15**	**75.4 ± 4.5**	**0.5**
		*OLC/LIGA*	*5/10*	*25.6 ± 4.2*	*0.9*
		OLC/OPA	5/10	55.3 ± 3.4	0.7
		**OLC/OM**	**5/25**	**71.9 ± 0.0**	**0.6**
		*LIGA/OPA*	*10/10*	*41.0 ± 5.7*	*0.9*

Values of inhibition of cell viability > 60% and CDIs < 1.0 (i.e., synergistic effect) are presented in bold (A) while values with lower cytotoxicity for the non-cancer cell lines are presented in italics (B).

**Table 2:** Selectivity indices (SI) of OOPs for skin tissue. The selectivity indices for the anti-proliferative/cytotoxic effect on skin cell lines were calculated by dividing the % inhibition caused by treatment with each double combination of OOPs for the non-cancer cell lines by those determined for the melanoma cell line A2058.

Selectivity Indices (SI)	
	RATIO HaCaT/A2058
OLC 10/OLA 30	1.3
OLC 10/LIG 35	1.0
OLC 10/OP 20	1.3
OLC 10/OM 35	1.0
OLA 30/LIG 35	1.1
OLA 30/OP 20	1.0
OLA 30/OM 35	1.2
LIG 35/OP 20	1.8
LIG 35/OM 35	1.1
OP 20/OM 35	1.2

**Table 3:** Anti-proliferative/cytotoxic activity of olive oil TPEs in cancer and non-cancer cell lines. The anti-proliferative/cytotoxic efficacy of olive oil TPEs was evaluated in (**A**) all the breast cancer (i.e., MDA-ΜΒ 231, SK-BR-3 και MCF-7) and melanoma cell lines (SK-MEL-28, A2058) available for this study and in the cell lines from each other cancer type studied herein which were less sensitive to the activity of the OOPs when used in the treatments as single compounds and (**B**) the non-cancer cell lines from breast (i.e., MCF 10A) and skin (i.e., HaCaT and NHDF). Treatments were performed as described in Materials and Methods section. Oleocanthal, OLC; oleacein, OLA; ligstroside aglycone, LIGA; oleuropein aglycone, OPA; oleomissional, OM; oleokoronal, OLK; tyrosol, TY.

**A.**	**EC_50_ Values**							
**Cell Lines**	**OOPs**	**Single OOP** **[20]**	**TPE I**		**TPE II**		**TPE III**	
		**μg/mL** **(±S.E.)**	**μΜ**	**μg/mL** **(±S.E.)**	**μΜ**	**μg/mL** **(±S.E.)**	**μΜ**	**μg/mL** **(±S.E.)**
**MDA-MB 231**	OLC	3.2 ± 0.2	12.5	3.8	13.1	4.0	0	0
	OLA	12.1 ± 0.7	5.2	1.7	5.4	1.7	0	0
	LIGA	11.5 ± 1.0	1.9	0.7	6.4	2.2	12.0	4.3
	OPA	9.6 ± 0.3	1.9	0.7	2.5	0.9	11.5	4.3
	OM	52.0 ± 7.6	0.0	0.0	1.1	0.4	1.4	0.5
	OLK	-	2.4	0.9	7.0	2.4	11.7	4.2
	TY	-	6.3	0.9	0.0	0.0	12.4	1.7
	Total Phenolic content			8.6 ± 1.2		11.5 ± 0.9		15.2 ± 0.5
**SK-BR-3**	OLC	3.9 ± 0.2	9.7	3.0	7.6	2.3	0	0
	OLA	14.6 ± 0.7	4.1	1.3	3.2	1.0	0	0
	LIGA	7.8 ± 0.9	1.5	0.5	3.7	1.3	7.9	2.9
	OPA	6.3 ± 0.6	1.5	0.5	1.5	0.5	2.9	2.9
	OM	20.2 ± 0.8	0.0	0.0	0.6	0.2	0.4	0.4
	OLK	-	1.9	0.6	4.1	1.4	2.8	2.8
	TY	-	4.9	1.2	0.0	0.0	1.1	1.1
	Total Phenolic content			7.0 ± 0.6		6.7 ± 0.6		10 ± 2.2
**MCF-7**	OLC	7.5 ± 0.8	22.3	6.8	16.9	5.1	0	0
	OLA	> 30	9.4	2.9	7.0	2.2	0	0
	LIGA	22.4 ± 0.4	3.3	1.1	8.3	2.8	18.0	6.5
	OPA	12.2 ± 0.4	3.3	1.1	3.2	1.1	17.3	6.5
	OM	> 30	0.0	0.0	1.4	0.5	2.2	0.8
	OLK	-	4.3	1.4	9.0	3.1	17.6	6.4
	TY	-	11.3	2.7	0.0	0.0	18.7	2.6
	Total Phenolic Content			16.0 ± 1.5		14.8 ± 0.7		22.9 ± 6.7
**SK-MEL-28**	OLC	3.2 ± 0.3	10.5	3.2	9.2	2.8	0	0
	OLA	10.7 ± 0.8	4.4	1.4	3.8	1.2	0	0
	LIGA	8.1 ± 0.4	1.6	0.5	4.5	1.6	7.3	2.7
	OPA	5.7 ± 0.3	1.6	0.5	1.8	0.7	7.0	2.7
	OM	17.1 ± 0.2	0.0	0.0	0.8	0.3	0.9	0.3
	OLK	-	2.0	0.7	4.9	1.8	7.2	2.6
	TY	-	5.3	1.2	0.0	0.0	7.6	1.1
	Total Phenolic content			7.5 ± 0.5		8.4 ± 0.9		9.3 ± 1.0
**A2058**	OLC	5.6 ± 0.1	11.6	3.5	10.0	3.0	0	0
	OLA	17.8 ± 1.1	4.9	1.5	4.2	1.3	0	0
	LIGA	23.1 ± 0.6	1.7	0.6	4.9	1.7	7.6	2.8
	OPA	14.1 ± 0.3	1.7	0.6	1.9	0.7	7.3	2.7
	OM	28.3 ± 0.5	0.0	0.0	0.8	0.3	0.9	0.3
	OLK	-	2.2	0.7	5.3	1.8	7.5	2.7
	TY	-	5.8	1.4	0.0	0.0	7.9	1.7
	Total Phenolic content			8.3 ± 0.7		8.8 ± 0.2		10.3 ± 1.3
**AGS**	OLC	5.6 ± 0.3	8.6	2.6	9.5	2.9	0	0
	OLA	14.8 ± 0.7	3.6	1.1	3.9	1.3	0	0
	LIGA	17.6 ± 0.9	1.3	0.4	4.6	1.7	7.7	2.8
	OPA	13.6 ± 0.5	1.3	0.4	1.8	0.7	7.4	2.8
	OM	28.5 ± 1.9	0.0	0.0	0.8	0.3	0.9	0.4
	OLK	-	1.6	0.6	5.0	1.8	7.6	2.7
	TY	-	4.3	0.9	0.0	0.0	8.0	1.1
	Total Phenolic content			6.1 ± 0.1		8.6 ± 0.4		9.8 ± 0.4
**HT-29**	OLC	8.0 ± 0.6	29.0	8.8	22.2	6.7		
	OLA	>30	12.2	3.8	9.2	3.0		
	LIGA	35.6 ± 3.1	4.3	1.4	10.9	3.9		
	OPA	18.9 ± 0.6	4.3	1.4	4.3	1.6		
	OM	>30	0.0	0.0	1.8	0.7		
	OLK	-	5.5	1.8	11.8	4.3		
	TY	-	14.6	3.5	0.0	0.0		
	Total Phenolic content			20.8 ± 5.4		20.2 ± 2.4		
**PANC-1**	OLC	4.5 ± 0.3	9.5	2.9	7.9	2.4		
	OLA	9.9 ± 0.1	4.0	1.3	3.3	1.0		
	LIGA	16.6 ± 0.5	1.4	0.5	3.9	1.4		
	OPA	7.2 ± 0.2	1.4	0.5	1.5	0.6		
	OM	13.2 ± 0.0	0.0	0.0	0.6	0.2		
	OLK	-	1.8	0.6	4.2	1.5		
	TY	-	4.8	1.1	0.0	0.0		
	Total Phenolic content			6.8 ± 0.7		7.2 ± 0.5		
**HepG2**	OLC	12.2 ± 1.6	18.0	5.5	8.2	2.5		
	OLA	26.5 ± 0.2	7.6	2.4	3.4	1.1		
	LIGA	16.6 ± 1.2	2.7	0.9	4.0	1.5		
	OPA	6.7 ± 0.2	2.7	0.9	1.6	0.6		
	OM	17.2 ± 1.3	0.0	0.0	0.7	0.3		
	OLK	-	3.4	1.1	4.4	1.6		
	TY	-	9.1	2.0	0.0	0.0		
	Total Phenolic content			12.8 ± 1.0		7.5 ± 0.4		
**Hela**	OLC	13.6 ± 0.1	26.5	8.1	60.0	18.2		
	OLA	14.8 ± 1.6	11.2	3.5	24.9	8.0		
	LIGA	10.5 ± 0.1	4.0	1.3	29.4	10.7		
	OPA	17.3 ± 0.6	4.0	1.4	11.5	4.4		
	OM	26.1 ± 0.6	0.0	0.0	4.9	1.9		
	OLK	-	5.0	1.7	31.9	11.6		
	TY	-	13.4	2.8	0.0	0.0		
	Total Phenolic content			18.8 ± 2.7		54.7 ± 8.2		
**H1299**	OLC	5.5 ± 0.3	15.7	4.8	10.4	3.2		
	OLA	19.8 ± 0.4	6.6	2.1	4.3	1.4		
	LIGA	30.2 ± 0.7	2.3	0.8	5.1	1.8		
	OPA	9.1 ± 0.3	2.3	0.8	2.0	0.8		
	OM	27.8 ± 1.7	0.0	0.0	0.9	0.3		
	OLK	-	3.0	1.0	5.5	2.0		
	TY	-	7.9	1.7	0.0	0.0		
	Total Phenolic Content			11.1 ± 0.5		9.5 ± 0.4		
**B.**		**Non—Cancer Cell Lines**						
**HaCaT**	OLC	5.9 ± 0.1	14.7	4.5	19.1	5.8		
	OLA	16.6 ± 1.4	6.2	1.9	7.9	2.5		
	LIGA	20.2 ± 0.4	2.2	0.7	9.4	3.4		
	OPA	9.2 ± 4.6	2.2	0.7	3.7	1.4		
	OM	16.1 ± 8.1	0.0	0.0	1.6	0.6		
	OLK	-	2.8	0.9	10.2	3.7		
	TY	-	7.4	1.8	0.0	0.0		
	Total Phenolic content			10.5 ± 2.5		17.4 ± 1.2		
**NHDF**	OLC	7.5 ± 0.1	18.1	5.5	16.4	5.0		
	OLA	15.7 ± 0.4	7.6	2.4	6.8	2.2		
	LIGA	16.9 ± 1.8	2.7	0.9	8.0	2.9		
	OPA	16.1 ± 0.2	2.7	0.9	3.1	1.2		
	OM	24.3 ± 0.7	0.0	0.0	1.3	0.5		
	OLK	-	3.4	1.1	8.7	3.2		
	TY	-	9.1	2.0	0.0	0.0		
	Total Phenolic content			12.9 ± 0.3		14.9 ± 0.6		
**MCF 10A**	OLC	2.1 ± 0.1	7.3	2.2	14.1	4.3		
	OLA	7.9 ± 0.9	3.1	1.0	5.8	1.9		
	LIGA	20.2 ± 1.0	1.1	0.4	6.9	2.5		
	OPA	3.6 ± 0.2	1.1	0.4	2.7	1.0		
	OM	13.4 ± 0.7	0.0	0.0	1.2	0.4		
	OLK	-	1.4	0.5	7.5	2.7		
	TY	-	3.7	0.8	0.0	0.0		
	Total Phenolic content			5.2 ± 0.5		12.8 ± 0.8		

Results presented are from two independent experiments performed in triplicates.

**Table 4:** Selectivity indices (SI) of olive oil TPEs for breast and skin tissues. SI values for the anti-proliferative/cytotoxic effect on breast and skin cell lines were calculated by dividing the EC_50_ values of TPEs I & II determined for the non-cancer cell lines by those determined for the cancer cell lines of corresponding tissue origin.

Cell Line/TPE	Selectivity Index (SI) (Control Cell Line)	
MDA-MB 231	(MCF 10A)	
TPE Ι	0.6	
TPE ΙΙ	1.1	
SK-BR-3	(MCF 10A)	
TPE Ι	0.7	
TPE ΙΙ	1.9	
MCF-7	(MCF 10A)	
TPE Ι	0.3	
TPE ΙΙ	0.9	
SK-MEL-28	(NHDF)	(HaCaT)
TPE Ι	1.5	1.2
TPE ΙΙ	1.8	2.1
A2058	(NHDF)	(HaCaT)
TPE Ι	1.5	1.3
TPE ΙΙ	1.7	2.0

**Table 5:** Comparison of EC_50_ values of olive oil TPEs with EC_50_ values of individual OOPs.

	MDA-MB 231	SK-BR-3	MCF-7	SK-MEL-28	A-2058	AGS	HT-29	PANC-1	HepG2	Hela	H1299
OOPs	**EC_50_ TPE I/EC_50_ OOP**										
OLC	2.7	1.8	2.1	2.4	1.5	1.1	2.6	1.5	1.1	1.4	2.0
OLA	0.7	0.5	0.5	0.7	0.5	0.4	0.6	0.7	0.5	1.3	0.6
LIGA	0.8	0.9	0.7	0.9	0.4	0.3	0.6	0.4	0.8	1.1	0.4
OPA	0.9	1.1	1.3	1.3	0.6	0.4	1.1	0.9	1.9	1.8	1.2
OM	0.4	0.3	0.3	0.4	0.3	0.2	0.5	0.5	0.7	0.7	0.4
	**EC_50_ TPE II/EC_50_ OOP**										
OLC	3.6	1.7	2.0	2.7	1.6	1.5	2.5	1.6	0.6	4.0	1.7
OLA	1.0	0.5	0.5	0.8	0.5	0.6	0.6	0.7	0.3	3.7	0.5
LIGA	1.1	0.9	0.7	1.0	0.4	0.5	0.6	0.4	0.4	3.2	0.3
OPA	1.2	1.1	1.2	1.5	0.6	0.6	1.1	1.0	1.1	5.2	1.0
OM	0.6	0.3	0.3	0.5	0.3	0.3	0.5	0.5	0.4	2.1	0.3
	**EC_50_ TPE III/EC_50_ OOP**										
OLC	4.8	2.5	3.1	2.9	1.8	1.7					
OLA	1.3	0.7	0.7	0.9	0.6	0.7					
LIGA	1.4	1.3	1.0	1.2	0.4	0.6					
OPA	1.6	1.6	1.9	1.6	0.7	0.7					
OM	0.8	0.5	0.5	0.5	0.4	0.4					

The ratio of EC_50_ values for the anti-proliferative/cytotoxic effect of olive oil TPEs and of single isolated OOPs was calculated based on the EC_50_ values (Table 3) of the five isolated secoiridoid derivatives evaluated by Papakonstantinou et al. 2022 [20]. The EC_50_ value of each extract was divided by the EC_50_ value of each isolated OOP for each cell line tested. Cases where the extracts were more effective (EC_50_ extract/EC_50_ OOP < 1) are highlighted in green and cases where the extracts were as effective as the single OOP (EC_50_ extract/EC_50_ OOP = 1) are highlighted in blue, while cases where the isolated single OOP was more effective (EC_50_ extract/EC_50_ OOP > 1) are highlighted in red. Oleocanthal, OLC; oleacein, OLA; ligstroside aglycone, LIGA; oleuropein aglycone, OPA; oleomissional, OM.

## Data Availability

Data are contained within the article or Supplementary Materials.

## Supplementary Materials

The following supporting information can be downloaded at: https://www.mdpi.com/article/10.3390/nu15112538/s1, Figure S1: Synergistic action of the best effective OOP double combinations [(A) oleacein with oleuropein aglycone; (B) oleacein with oleomissional and (C) oleacein with ligstroside aglycone] on the best responded to treatment human cancer cell lines; Figure S2: Synergistic action of OOP double combinations on the best responded to treatment AGS, Panc-1 and H1299 human cancer cells; Table S1: Chemical structures of OOPs used in this study; Table S2: List of the cancer and non-cancer cell lines used in the study; Table S3: Total Phenolic Extracts (TPEs) from olive oil. OOP compositions in the extracts used in this study.
