# Supplementary material for: A Systematic Ex-Vivo Study of the Anti-Proliferative/Cytotoxic Bioactivity of Major Olive Secoiridoids’ Double Combinations and of Total Olive Oil Phenolic Extracts on Multiple Cell-Culture Based Cancer Models Highlights Synergistic Effects
Source: Nutrients. 2023 May 29;15(11):2538. doi: 10.3390/nu15112538 (PMC10255584; doi:10.3390/nu15112538)
Supplement: Supplementary file 1 [file nutrients-15-02538-s001.zip › nutrients-2404820-supplementary.pdf]

Supplementary material for:

A systematic ex-vivo study of the anti-proliferative / cytotoxic bioactivity of Major Olive Secoiridoids' Double Combinations and of Total Olive Oil Phenolic Extracts on multiple cell-culture based cancer models highlights Synergistic Effects

Aikaterini Papakonstantinou[†1,2], Petrina Koumarianou[†1], Panagiotis Diamantakos[2], Eleni Melliou[2,3], Prokopios Magiatis[2*], Haralabia Boleti[1*]

[1] Intracellular Parasitism Laboratory, Microbiology Department, Hellenic Pasteur Institute, 115 21, Athens, Greece

[2] Laboratory of Pharmacognosy and Natural Products Chemistry, Department of Pharmacy, National and Kapodistrian University of Athens, Panepistimioupolis Zografou, 15771 Athens, Greece

[3] World Olive Center for Health, Imittou 76, 11634 Athens, Greece

Table of contents

Figure S1. Synergistic action of the best effective OOPs' double combinations [A) oleacein with oleuropein aglycone; B) oleacein with oleomissional and C) oleacein with ligstroside aglycone] on the best responded to treatment human cancer cell lines.

Figure S2. Synergistic action of OOPs' double combinations on the best responded to treatment AGS, Panc-1 and H1299 human cancer cells.

Table S1. Chemical structures of OOPs used in this study

Table S2. List of the cancer and non-cancer cell lines used in the study

Table S3. Total Phenolic Extracts (TPEs) from olive oil. OOPs' compositions in the extracts used in this study

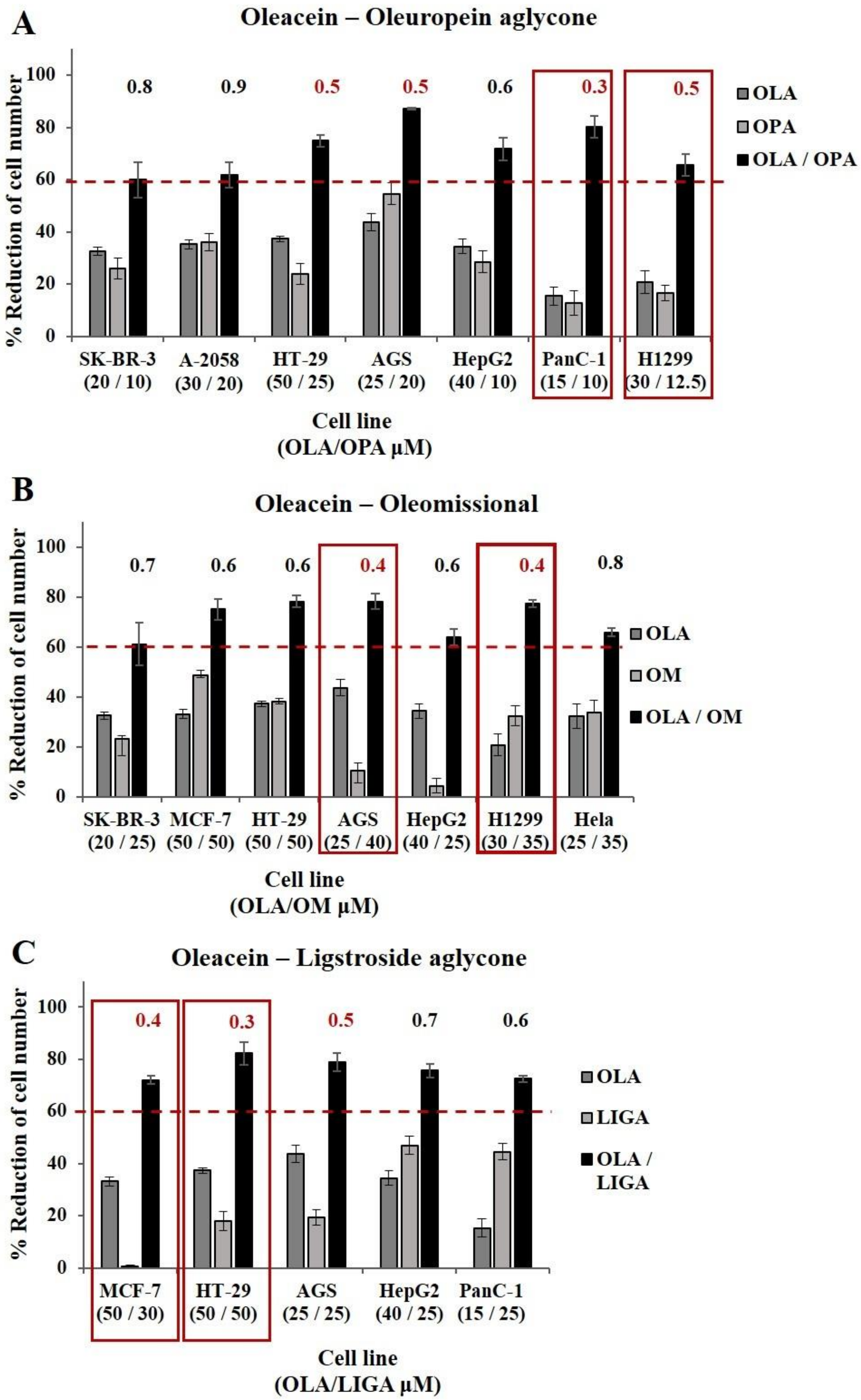

Figure S1. Synergistic action of the best effective OOPs' double combinations [A) oleacein with oleuropein aglycone; B) oleacein with oleomissional and C) oleacein with ligstroside aglycone] on the best responded to treatment human cancer cell lines.

The viability of the SK-BR-3, MCF-7, A-2058, HT-29, AGS, HEPG-2, Panc-1 and H1299 human cancer cells was evaluated using the MTT assay after 72 h treatment with double combinations at concentrations ½ EC_{50} value of each for the tested cell line. The effectiveness in reducing cell numbers of each single compound used in the combination at the ½ EC_{50} value was evaluated in parallel and plotted next to the double combination results from two independent experiments performed in triplicates. Only the results of treatments that resulted to 60% reduction of cell numbers in the double combinations are presented. Data are mean cell counts ± SE in each treatment group normalized to the control group (cells treated only with 0.2% (v/v) DMSO). CDI values for each combination were calculated and presented on top of each bar.

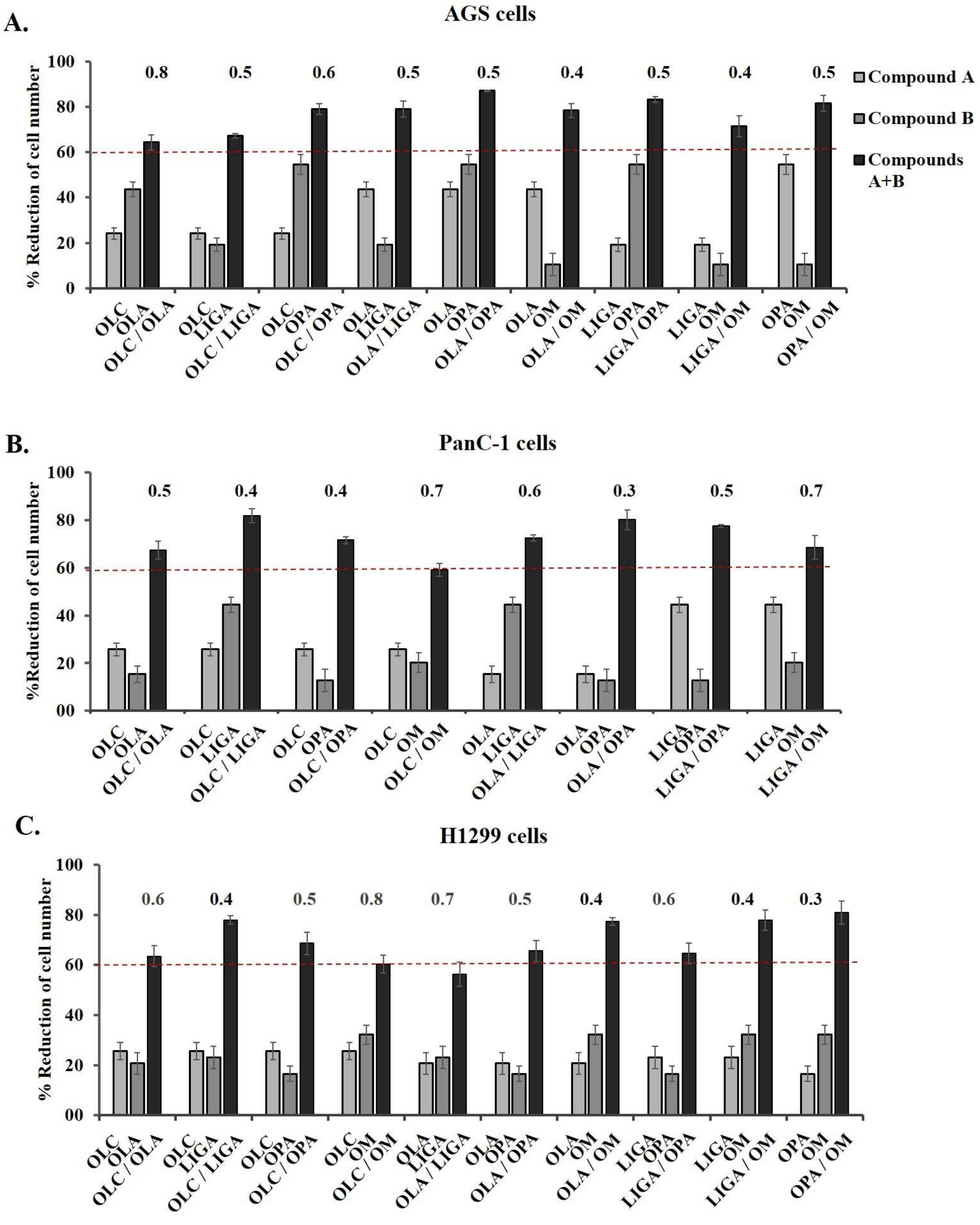

Figure S2. Synergistic action of OOPs' double combinations on the best responded to treatment AGS, Panc-1 and H1299 human cancer cells. The viability of the AGS (A), Panc-1 (B) and H1299 (C) human cancer cells was evaluated using the MTT assay after 72

h treatment with 8-10 different double combinations of five different OOPs at concentrations ½ EC_{50} value of each for the tested cell line. The effectiveness in reducing cell numbers of each single compound used in the combination at the ½ EC_{50} value was evaluated in parallel and plotted next to the double combination results from two independent experiments performed in triplicates. Data are mean cell counts ± SE in each treatment group normalized to the control group (cells treated only with 0.2% (v/v) DMSO). CDI values for each combination were calculated and presented on top of each bar.

Table S1. Chemical structures of OOPs used in this study

Name	Abbreviation	Molecular Structure
Oleocanthal (p-HPEA-EDA)	OLC	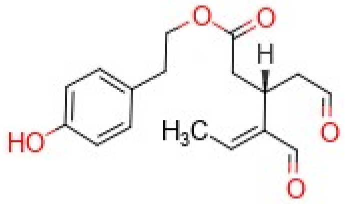
Oleacein (3,4, DHPEA-EDA)	OLA	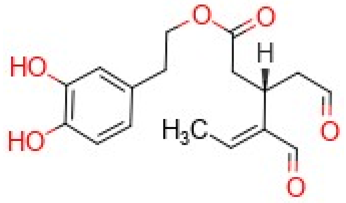
Oleomissional	OM	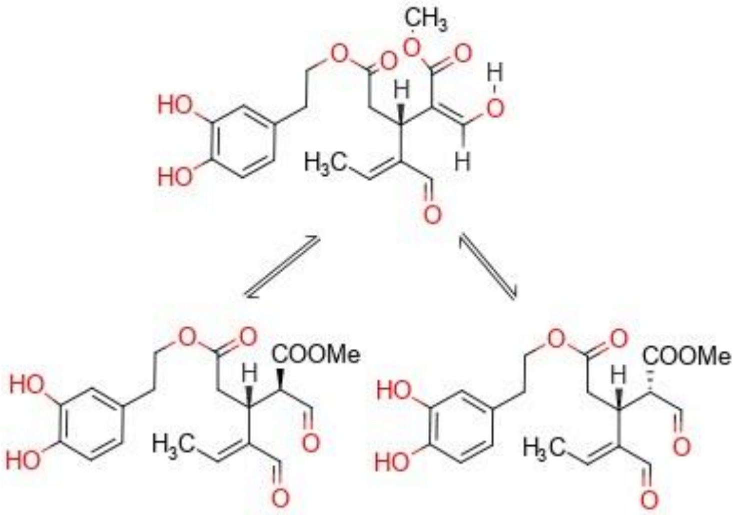
Oleokoronal	OLK	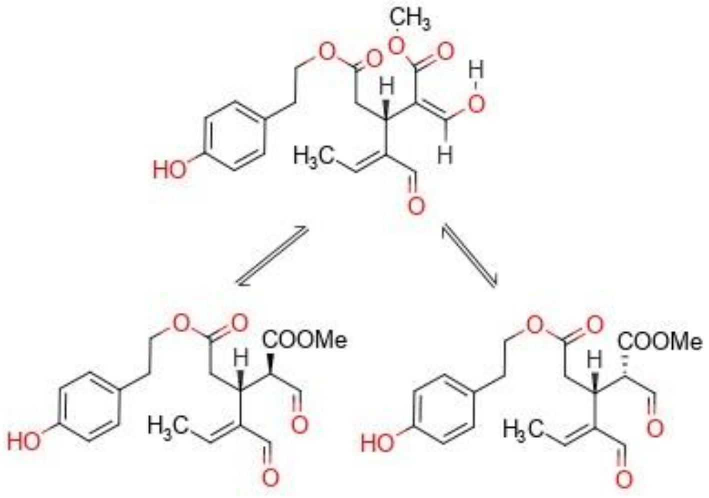

Oleuropein aglycone-closed type (3,4, DHPEA-EA)	OPA	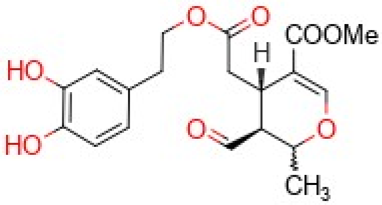
Ligstroside aglycone-closed type (p-HPEA-EA)	LIGA	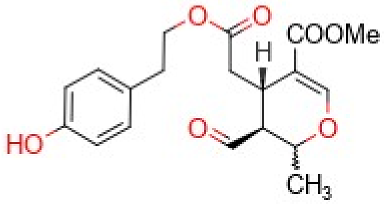
Tyrosol	TY	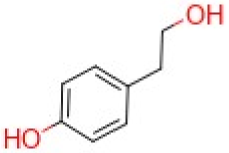

Table S2. List of the cancer and non-cancer cell lines used in the study

CELL LINE NAME	CANCER CELL MODEL (Tissue type tumor origin and characteristics)	SOURCE
	**COLON**	
HT-29 (ATCC-HTB-38)	Human colon adenocarcinoma; CD4 negative; cell surface expression of galactose ceramide; The p53 antigen is overproduced, and there is a G → A mutation in codon 273 of the p53 gene resulting in an Arg → His substitution.	Provided by Dr. Sgouras, Hellenic Pasteur Institute, Athens, Greece.
	**STOMACH**	
AGS (ATCC-CRL-1739)	Human gastric cancer cell line	Provided by Dr. Sgouras, Hellenic Pasteur Institute, Athens, Greece.
	**LIVER**	
HepG-2	Epithelial like, hepatocellular carcinoma	Provided by Prof. G. Notas, University of Crete, School of Medicine, Heraclion, Crete, Greece. (gnotas@uoc.gr)
	**PANCREAS**	
PANC-1 (ATCC- CRL-1469)	Pancreatic ductal adenocarcinoma cell line (Epithelioid Carcinoma)	Provided by Dr I. Papasotiriou, RGCC International GmbH (office@rgcc-international.com)
	**BREAST**	
SK-BR-3 (ATCC® HTB-30)	Mammary gland breast adenocarcinoma; HER2 +ve. : ER-, PR-, HER2+	Purchased from ATCC
MCF-7 (ATCC- HTB-22)	Mammary gland, breast cancer adenocarcinoma: ER+, PR+/-, HER2- (slowly growing epithelial cells)	Provided by Dr. Kletsas, Institute of Biosciences and Applications, NCSR Democritus
MDA-MB-231 (ATCC- HTB-26)	Breast, mammary gland adenocarcinoma epithelial cells. Triple negative (i.e. ER^-, PR^-, $HER2^-$)	Provided by Dr. Lymberi, Immunology laboratory, Hellenic Pasteur Institute, Athens, Greece
	**LUNG**	
H1299 (NCI H1299) (ATCC CRL-5803)	Non-small Lung cancer;derived from metastatic site: lymph node, p53 deficient	Provided by Prof. E. Kolettas, Biology Lab, University of Ioannina Medical School & IMBB, Ioannina, Greece
	**SKIN**	
A2058 (CRL-11147)	Human melanoma; epithelial cells from skin: derived from metastatic site: lymph node	Purchased from ATCC
SK-MEL-28	Melanocytes; Human Malignant Melanoma	Purchased from ATCC

(HTB-72)		
	**CERVIX**	
Hela (ATCC- CCL-2)	Human cervix adenocarcinoma with low P53 expression and normal levels of pRB (retinoblastoma suppressor).	Kind gift from Dr A. Dautry, Laboratory of Biologie des Interactions cellulaires, Institut Pasteur, Paris, France
	**NON CANCER CELLS**	
NHDF (PCS-201-012)	Normal Adult Human Primary Dermal Fibroblasts isolated from the dermis of juvenile foreskin or adult skin.	Provided by Dr. Sophia Letsiou, APIVITA R&D department, Athens, Greece
HaCaT (CLS - 300493)	Spontaneously transformed aneuploid immortal keratinocyte cell line from adult human skin	Purchased from CLS cell line service
MCF 10A (ATCC-CRL-10317)	Epithelial Human mammary gland; breast	Purchased from ATCC

Table S3. Total Phenolic Extracts (TPEs) from olive oil. OPPs' compositions in the extracts used in this study

Compounds	Concentration (mM)	Concentration (mg/mL)	% of total phenolic content
		**TPE I**	
Oleocanthal	53.6	16.6	44.6
Oleacein	22.6	7.2	19.4
Ligstroside Aglycone	8	3	8.1
Oleuropein Aglycone	8	3	8.1
Oleomissional	0	0	0
Oleokoronal	10.2	3.7	9.9
Tyrosol	27	3.7	9.9
		**TPE II**	
Oleocanthal	50	15.2	33.9
Oleacein	20.8	6.3	14.1
Ligstroside Aglycone	24.5	8.8	19.6
Oleuropein Aglycone	9.6	3.5	7.8
Oleomissional	4.1	1.5	3.3
Oleokoronal	26.6	9.5	21.2
Tyrosol	0	0	0
		**TPE III**	
Oleocanthal	0	0	0
Oleacein	0	0	0
Ligstroside Aglycone	50	18.1	28.5
Oleuropein Aglycone	48	18.2	28.6
Oleomissional	6	2.3	3.6
Oleokoronal	49	17.8	28.0
Tyrosol	52	7.2	11.3
